# Parallel-Channel PIV System for Time-Resolved Measurement of Shock Wave Reflection and Diffraction

**DOI:** 10.3390/s26154866

**Published:** 2026-08-02

**Authors:** Tianqing Zhao, Zutang Wu, Jun Yang, Junyi Guan, Jin Li, Guoliang Li

**Affiliations:** National Key Laboratory of Intense Pulsed Radiation Simulation and Effect, Northwest Institute of Nuclear Technology, Xi’an 710024, China; zhaotianqing@nint.ac.cn (T.Z.); lijin@nint.ac.cn (J.L.);

**Keywords:** particle image velocimetry, shock wave diffraction, multi-channel parallel system, time-resolved measurement, compressible flow

## Abstract

Conventional particle image velocimetry (PIV) techniques face an inherent trade-off between temporal resolution and single-pulse laser energy, limiting their performance in shock wave measurements. This study develops a multi-channel parallel PIV system with programmable timing control to address this constraint. By distributing laser pulses across eight independent optical channels, the system decouples single-pulse energy from the repetition rate, enabling continuous velocity field measurements at microsecond temporal resolution with high signal-to-noise ratio. Experiments were conducted on a planar shock wave propagating over a trapezoidal step in a shock tube facility. The system captured velocity fields at seven consecutive time instants with a 1 μs pulse interval, revealing the curved evolution of diffracted shock fronts and transient flow separation induced by shock–boundary-layer interaction. Comparison with unsteady Reynolds-averaged Navier–Stokes simulations demonstrated that the PIV system resolves fine-scale post-shock perturbations and turbulent fluctuations that were numerically dissipated in computational fluid dynamics. The results verify that the parallel-channel architecture accurately captures unsteady flow structures during shock reflection and diffraction, offering a reliable diagnostic technique for investigations of shock wave dynamics.

## 1. Introduction

Shock waves widely exist in military engineering, industrial safety, aerospace applications and disaster prevention. Their impacts become prominent in high-energy-release events, including nuclear and chemical explosions. Thermodynamic parameters change abruptly across a shock front, forming strongly unsteady and spatially nonuniform post-shock flows [1]. For practical applications, protective devices such as blast helmets have been developed to suppress the destructive effects of shock waves [2]. Meanwhile, the high energy carried by shock waves is utilized in advanced propulsion systems, e.g., pulse detonation engines [3]. Accurate characterization of the evolution of complex wave systems, especially for high-speed shock interactions with irregular geometries, is essential for the optimization of the above engineering technologies.

Shock propagation over complex boundaries has attracted considerable research attention due to its importance in energy transfer analysis and structural damage prediction. Complex shock reflection and diffraction generally occur when shocks propagate in confined domains or across geometric discontinuities. Traditional diagnostic techniques, including schlieren imaging and pressure transducers, have been widely adopted to investigate macroscopic wave structures [4,5,6,7]. However, most existing analytical models are built upon quasi-steady assumptions. Practical engineering scenarios, such as blast loading, supersonic inlet flows and detonation-driven flows, feature highly unsteady flow behaviors dominated by the strong coupling between transient wave systems and vortical structures. When a planar shock impinges on complex geometries, the post-shock flow involves wave reflection, wave superposition and intense shock diffraction. Resolving the fine flow features during shock diffraction remains a major challenge for experimental measurements, which requires full-field spatial information and microsecond-scale temporal resolution simultaneously.

Unlike well-documented reflected wave patterns, the shock diffraction region behind obstacles exhibits strong spatiotemporal complexity caused by viscous–inviscid interactions and vortex entrainment. Investigating the formation, evolution and interaction of transient vortical structures in this region is critical to understanding unsteady compressible turbulence, while this task still faces great difficulties. Conventional single-point sensors (e.g., pressure transducers) cannot provide full-field spatial data, and line-of-sight optical methods such as shadowgraph and schlieren techniques are only capable of qualitative flow visualization. These methods are unable to resolve three-dimensional transient vorticity fields or acquire instantaneous velocity data for scale-resolved turbulence analysis [8,9,10]. As a non-intrusive experimental technique, particle image velocimetry (PIV) has become a powerful tool in modern fluid mechanics. It provides full-field and quantitative velocity measurements. With double-pulsed laser illumination and cross-correlation algorithms, PIV can acquire velocity vectors at thousands of spatial locations in a planar domain, enabling the characterization of multiscale flow topologies and the quantification of energy cascades from integral scales down to the measurement limit [11,12,13,14].

When applied to highly transient phenomena such as shock wave diffraction, existing PIV techniques still face significant limitations. Since the diffraction region encompasses both multiscale complex flow structures and shock fronts with sharp spatial gradients, conventional PIV is often constrained by the field of view, temporal resolution, and image signal-to-noise ratio (SNR) achievable in a single acquisition, making it difficult to obtain high-resolution, time-synchronized measurements of vortex dynamics in the diffraction region [15,16,17]. Therefore, overcoming the spatiotemporal resolution constraints of conventional PIV in transient, large-scale flow field measurements is essential for elucidating the underlying physical mechanisms of shock diffraction.

Dynamic measurements of transient shock phenomena require the system to balance two competing demands: mitigating motion blur through nanosecond-scale exposure (which demands high single-pulse energy) and maintaining high acquisition rates for time-resolved characterization [18,19,20]. Current research efforts have advanced along two fronts. At the algorithmic level, deep learning-based denoising and robust cross-correlation methods have been developed to extract velocity information from low-SNR images [21,22,23]; however, algorithms cannot compensate for the fundamental lack of physical photon signals. At the hardware level, pulse-burst PIV systems coupling high-repetition-rate lasers with high-speed CMOS cameras achieve acquisition rates of ~100 kHz over burst durations of approximately 100 ms [20,24]. Miller et al. demonstrated that this technology successfully captured the transient evolution of shock–boundary layer interaction (SBLI) [24].

In strongly unsteady flow fields characterized by near-discontinuous gradients in thermodynamic properties, such as shock waves, such systems still fail to provide sufficient illumination intensity. This deficiency is particularly acute in the post-shock region, where the passage of the shock front depletes tracer particles and dramatically reduces the particle scattering density available for cross-correlation analysis [12,25]. This stems from a fundamental physical limitation: single-source high-repetition-rate lasers inevitably suffer from a sharp reduction in per-pulse energy as the frequency increases. Consequently, such systems cannot simultaneously provide the high pulse energy required for shock-front imaging and the sustained SNR needed for time-resolved sequences, rendering the simultaneous high-resolution spatiotemporal characterization of the entire flow field a significant challenge.

To address this, this paper proposes a parallel-channel PIV architecture. This architecture employs a sequential multi-channel triggering strategy to synchronize multiple high-energy Nd:YAG lasers with high-resolution cameras. By distributing the pulse emission across parallel optical channels, this design decouples the single-pulse energy from the system level repetition rate. Importantly, the high single-pulse energy (400 mJ at λ = 532 nm) characteristic of conventional low-repetition-rate PIV systems (≤15 Hz) is retained. This high pulse energy ensures adequate illumination intensity even under nanosecond-scale exposures, enabling high-SNR imaging of tracer particles, while also effectively freezing the motion of high-speed particles near shock fronts to eliminate particle streaking. Through precisely timed multi-channel triggering, this architecture achieves microsecond temporal resolution, effectively overcoming the conventional trade-off between pulse energy and temporal resolution in shock wave diagnostics.

This study leverages the high-fidelity capabilities of the developed multi-channel parallel PIV system to investigate the spatiotemporal evolution of energy and vortical dynamics in shock-driven flows. Specifically, experiments were conducted on a planar shock wave propagating over a trapezoidal step, with a particular focus on the synchronized characterization of diffracted shock front evolution and the subsequent development of shear-layer instabilities. The experimental results successfully capture high-resolution evolutionary sequences of the transient flow field, providing quantitative insights into the formation and evolution of vortical topologies on the leeward side, as well as the identification of distinct recirculation zones after the shock wave passes over the step.

## 2. Materials and Methods

### 2.1. Experimental Setup

**Figure 1:**
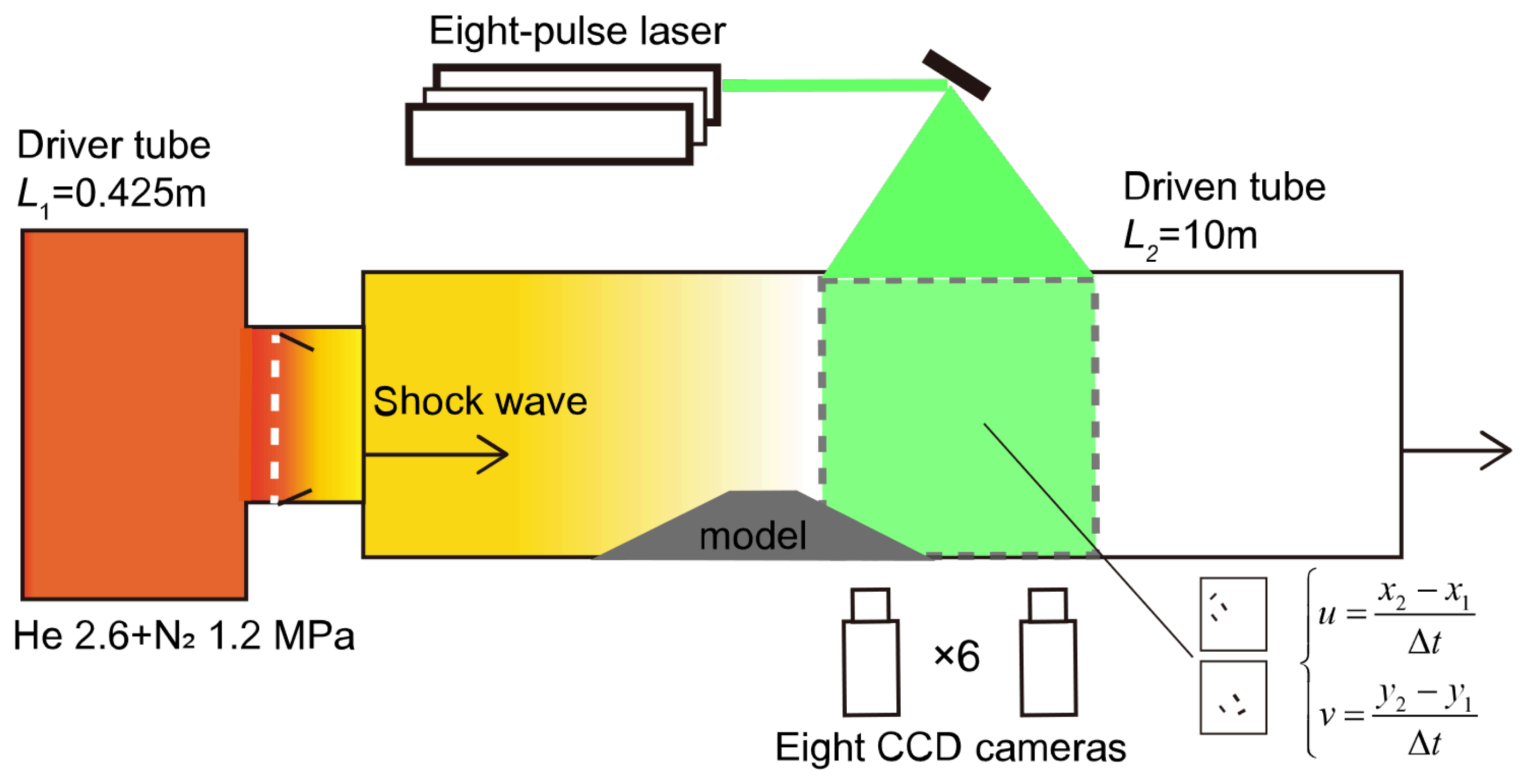
Schematic diagram of the experimental setup.

All experiments were conducted in a horizontal shock tube facility with a driver section 425 mm in length, featuring an inner diameter converging from 100 mm to 48 mm, yielding a cross-sectional area contraction ratio of 4.34. A high-pressure gas mixture, with a total pressure of approximately 3.8 MPa, was employed as the driver gas. The driven section consists of a square-cross-section channel with an inner side length of 234 mm (corresponding to an area-equivalent diameter of 264 mm), extending to a total length of 10 m with a closed end. To capture the transient interactions, the PIV test section is equipped with an observation window located 1.75 m downstream of the diaphragm, providing an axial field of view of 265 mm, as shown in Figure 1.

A light-sheet optical system transforms the combined laser beams into a thin sheet approximately 1 mm in thickness, aligned parallel to the flow direction. A cylindrical lens (*f* = 150 mm) expanded the combined laser beams to form a light sheet approximately 1 mm thick, verified by photosensitive paper measurement. Alignment to the streamwise centerplane was achieved using a high-precision calibration target. The data acquisition sequence is initiated by the incident shock wave; a pressure transducer located 350 mm upstream of the observation window detects the wave arrival, providing the master trigger signal for the laser and camera systems.

**Figure 2:**
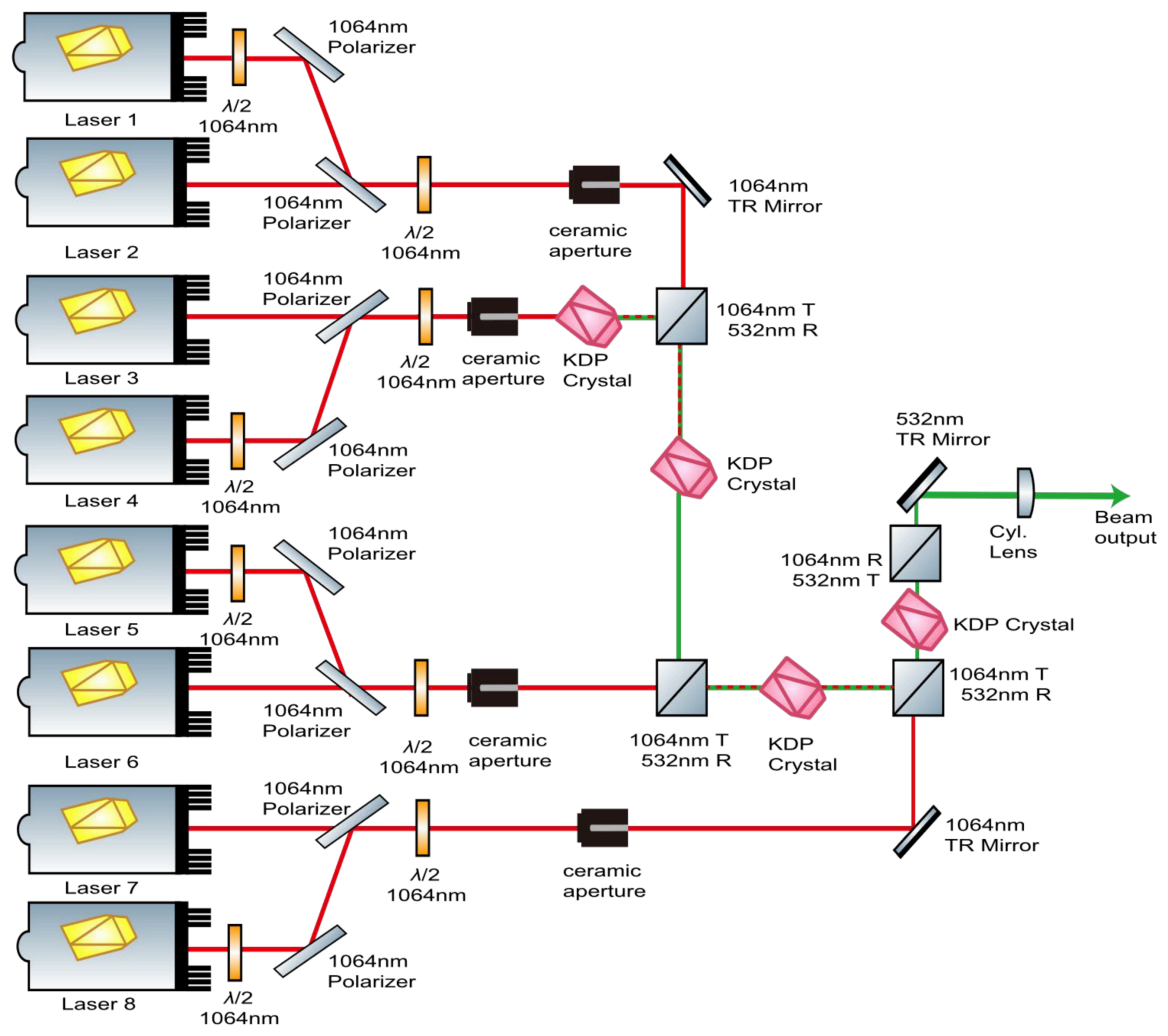
Optical path diagram of the 8-cavity laser light source combiner.

As illustrated in Figure 2, a staged grouping optical architecture was implemented. Beams are paired at 1064 nm prior to frequency doubling, minimizing green-optic damage risks and distributing thermal load across multiple KDP crystals to avoid the transient thermal lensing inherent to single-crystal configurations.

A programmable synchronization controller serves as the central timing hub, coordinating the operation of the eight lasers and digital cameras. Using the pressure sensor signal as the external timing reference, the controller outputs multiple TTL signals with independent, programmable delays. The firing sequence of the eight laser pulses and camera exposure windows is synchronized by adjusting channel delays. Each channel delay is independently programmable, enabling either uniform pulse separation or customized non-uniform temporal spacing. This timing architecture ensures a precision of ±1 ns. This timing architecture is controlled by a custom FPGA-based synchronizer with a nominal ±1 ns channel-to-channel jitter, validated experimentally using a high-bandwidth oscilloscope with fast photodetectors.

**Figure 3:**
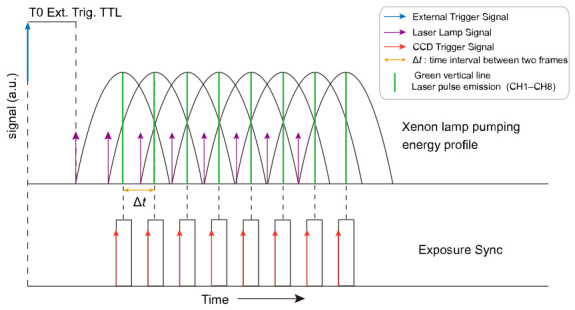
System synchronization timing diagram.

The complete timing sequence of the PIV system is illustrated in Figure 3. Specifically, the system operates in a sequential single-pulse, single-frame mode. The synchronizer coordinates the sequential firing of eight independent laser pulses, each matched to the exposure window of one corresponding CCD camera. This yields eight independent single-exposure particle images at preset intervals, which are cross-correlated in consecutive pairs to reconstruct seven time-resolved velocity fields.

### 2.2. Measurement Principle

**Figure 4:**
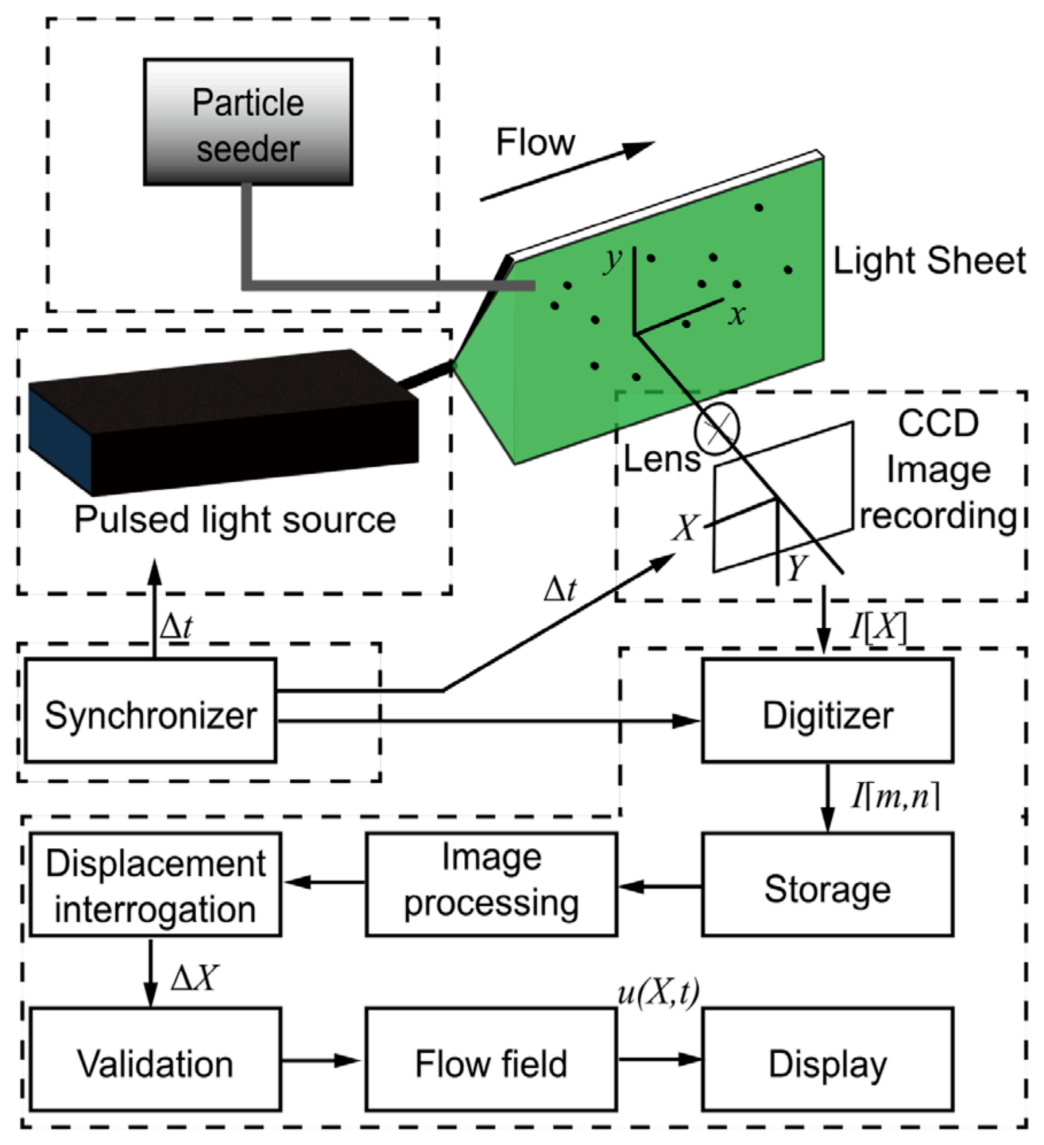
PIV System composition and velocimetry principle.

Figure 4 summarizes the parallel-channel PIV measurement principle. Tracer particles are introduced into the flow field, where they are illuminated by the 8-pulse Nd:YAG laser. In contrast to conventional single-channel PIV, this architecture utilizes a multi-channel timing strategy managed by a master synchronizer. This setup enables precise coordination between the firing of each laser pulse and the exposure window of its corresponding CCD camera with ns accuracy. By distributing the image acquisition across eight parallel channels, the system records a sequence of eight high-fidelity particle images at preset intervals, effectively capturing the time-resolved evolution of the transient flow field.

### 2.3. Image Processing and Uncertainty Analysis

In this study, PIV velocity field measurements were performed using a multi-pass cross-correlation algorithm with window deformation. Prior to velocity calculation, all raw particle images were pre-processed via background subtraction and intensity normalization to eliminate image noise and improve data reliability. A two-pass cross-correlation evaluation strategy was adopted for PIV interrogation, with the interrogation window size gradually refined from 64 × 64 pixels to 32 × 32 pixels and a 50% window overlap rate applied. The window deformation technique was implemented to adapt to the strong shear flow characteristics of the measured field and capture complex flow variations accurately.

To guarantee the validity of velocity vector data, systematic post-processing was conducted after velocity field calculation. A normalized median test (threshold Q < 1.2) was used to identify and replace spurious erroneous vectors. Meanwhile, the pulse separation time (Δ*t*) was dynamically adjusted from 1 μs to 8 μs according to regional flow velocity differences, ensuring particle displacement remained within the optimal range of 5–10 pixels across the entire measurement field. The established PIV measurement system achieved a final spatial resolution of approximately 3 mm per vector.

A quantitative uncertainty budget was further established to evaluate measurement accuracy, with all dominant error sources systematically analyzed. First, the timing synchronization precision of ±1 ns introduced a relative velocity uncertainty of less than 0.1% for the measured shock propagation velocity of approximately 443 m/s. Second, spatial calibration was performed using a high-precision target, yielding a calibration factor of 0.0845 mm/pixel with a corresponding uncertainty of ±0.5%. Third, the constrained optimal particle displacement (5–10 pixels) limited the subpixel interpolation error to approximately 0.1 pixels, as reported in previous canonical studies [26]. Fourth, ensemble averaging over multiple repeated experimental runs was adopted to eliminate the interference of shot-to-shot shock overpressure fluctuations (variation of ±20%).

By synthesizing all independent error sources in quadrature, the total relative measurement uncertainty of the present PIV test system was estimated to be less than 2%. This accuracy level is consistent with the high-precision PIV uncertainty range summarized in classic research [25] and matches the theoretical precision analysis results of PIV measurement proposed by Westerweel [27], verifying the reliability and accuracy of the experimental measurement data.

The main parameters of the parallel-channel PIV system are detailed in Table 1.

## 3. Results

Based on the aforementioned parallel-channel PIV system, this study measured the interaction between a planar shock wave and a trapezoidal model in a Ø264 mm shock tube. Due to the limited test time in the shock tube and the transient nature of the flow evolution, PIV measurements were performed at two characteristic time points following diaphragm rupture (*t* = 4 ms and *t* = 8 ms) in separate experimental runs by varying the trigger delay. At *t* = 4 ms, the shock front traverses the observation window, representing the shock-passage phase. At *t* = 8 ms, the shock wave has completely swept across the window and the vortex structure is fully developed, representing the vortex-dominated phase. This approach to reconstructing the temporal evolution is valid due to the highly repeatable nature of the shock tube flow.

**Figure 5:**
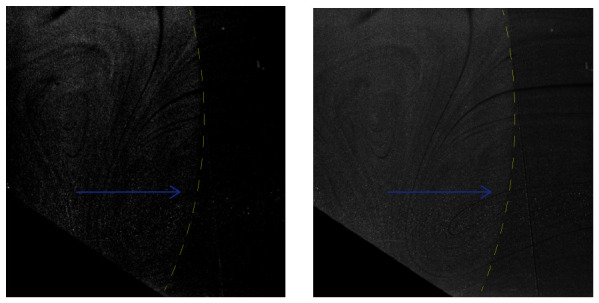
Raw particle images recorded by PIV. The arrows indicate the flow direction, and the dashed lines denote the shock wavefronts.

Figure 5 exhibits the raw particle image sequence captured by the parallel-channel PIV system at Mach 1.3, depicting a planar shock wave diffracting over a trapezoidal model. The incident shock front, characterized by a high-contrast density jump, is clearly visible. The high single-pulse energy and short pulse duration (<10 ns) effectively prevent motion blur. This ensures a high signal-to-noise ratio in the particle images, demonstrating the system’s excellent temporal control and imaging performance.

### 3.1. Shock Wave Propagation and Macroscopic Wave Structure

**Figure 6:**
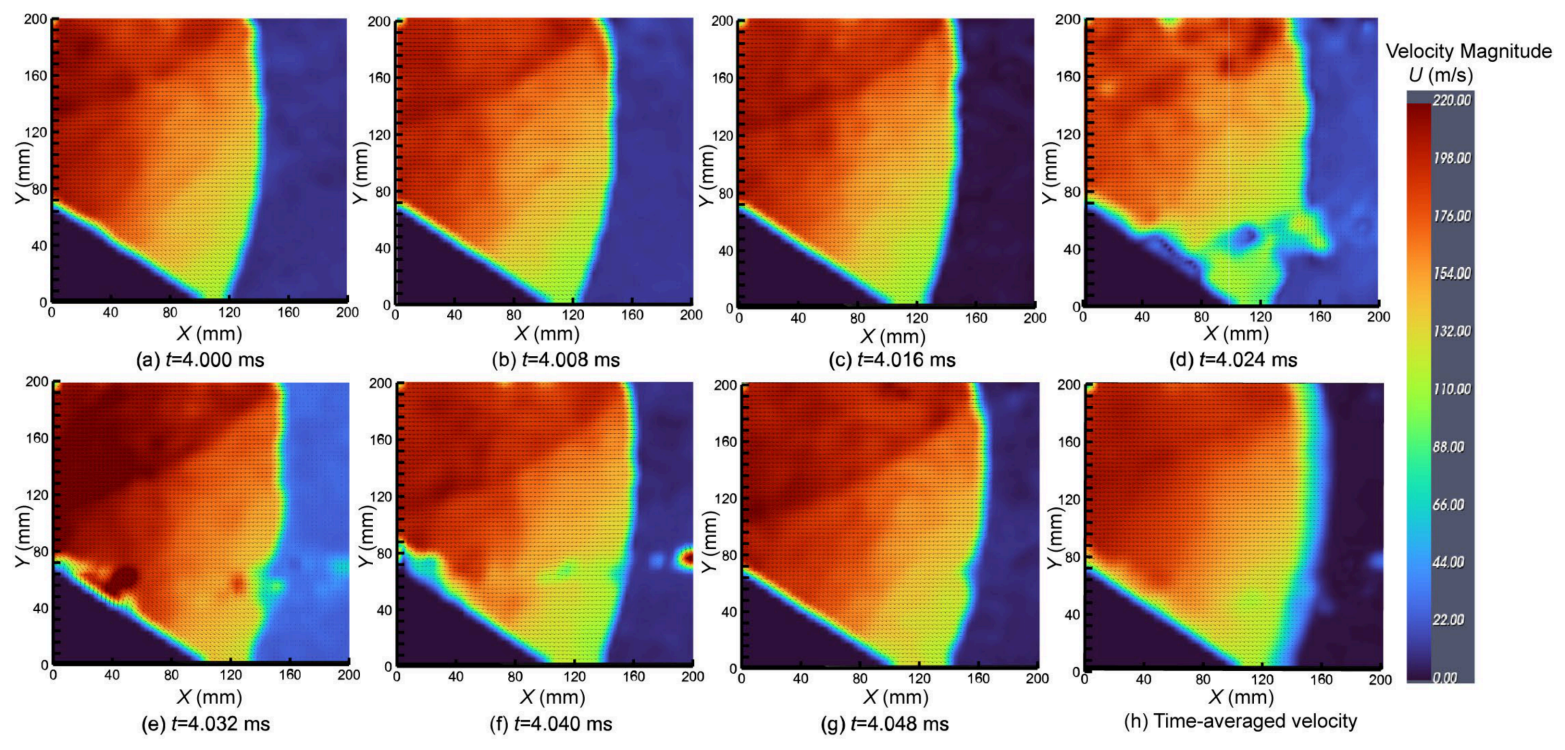
Instantaneous velocity field 4 ms after diaphragm rupture. The color contours represent the velocity magnitude $U=\sqrt{u^{2}+v^{2}}$ m/s.

Figure 6 exhibits the sequence of instantaneous velocity fields at t = 4 ms (Figure 6a–g, Δ*t* = 8 μs) and the ensemble-averaged velocity field (Figure 6h), both obtained employing the parallel-channel PIV system. This instant marks the critical stage of the shock front traversal across the observation window. The planar shock has just passed the leeward side of the trapezoidal model, and the post-shock flow is in its early development phase.

The time-resolved results exhibit that the incident shock diffracts after passing the model corner, leading to significant curvature of the shock front. Observations at 8 μs intervals indicate that the wavefront shape and curvature distribution remain highly consistent between adjacent frames, confirming that the wavefront curvature has reached a quasi-steady state. The ensemble-averaged velocity field (Figure 6h) clearly reveals the spatial inhomogeneity of the flow. A strong velocity contrast exists between the high-speed post-shock main flow in the upper-left region and the low-speed recirculation zone in the lower-middle area. Furthermore, the reflected shock wave from the upper-left wall induces a distinct velocity discontinuity. The flow evolution exhibits that this discontinuity propagates from the upper left to the lower right, undergoing distortion as the vortical structures evolve.

The shock wave propagation velocity was quantified via wavefront tracking. In the instantaneous velocity field sequence at *t* = 4 ms, the shock front was identified by detecting regions with high velocity gradients where the velocity jump Δ*u* ≥ 100 m/s. As shown in Figure 6, the extracted shock front contours are clear and highly consistent across consecutive frames. The instantaneous propagation velocity is calculated employing Equation (1):

(1) $$V=\frac{∆s\cdot M}{∆t}$$

where *V* is the instantaneous shock propagation velocity (m/s), Δ*s* represents the displacement of the shock front in pixels detected between consecutive frames, *M* is the spatial calibration factor (mm/pixel), and Δ*t* is the programmed temporal interval between the two corresponding laser pulses (s).

To suppress random errors in single-shot measurements, a three-point moving average was applied to data from three consecutive frames, ultimately yielding an average shock wave propagation velocity of *V_avg_* = 443 ± 12 m/s (mean ± standard deviation) [28].

The aforementioned wavefront bending implies a coupling mechanism between shock wave dynamics and the resulting flow field. When a planar shock wave propagates toward the trapezoidal model, a high-pressure region forms on the windward side. As the shock diffracts at the model edge, the post-shock flow undergoes an expansion process, resulting in a non-uniform shock velocity distribution along the wavefront normal. Specifically, the shock velocity decreases near the model due to the stagnation effect, whereas the region far from the model maintains its original propagation velocity. This velocity gradient disrupts the planarity of the shock wave; according to Whitham’s ray-shock theory, this gradient governs the shock front curvature, causing the wavefront to bend toward the leeward side. Subsequently, as the expansion waves propagate and the diffracting shock system adapts to the convex geometry, a dynamic equilibrium is established in the wave structure. This self-similar evolution suppresses instantaneous wavefront fluctuations, allowing the shock front to enter a pseudo-steady state.

The wavefront bending observed at *t* = 4 ms in this study is qualitatively consistent with Skews’ classic experimental results. Skews employed schlieren photography (with a frame interval of ~0.5 ms) to systematically investigate the diffraction of a planar shock wave at a convex corner. He found that the diffracted shock front bends and exhibits pseudo-steady characteristics within the observation time; that is, the curved shock shape and the disturbance region remain geometrically similar over time. Both studies demonstrate that convex geometries induce wavefront bending in planar shock waves, and this bending exhibits pseudo-steady characteristics once the shock crosses the leeward side.

It is important to emphasize that Skews’ classic investigations rely on schlieren photography, a line-of-sight-integrated measurement technique. By contrast, the parallel-channel PIV system developed herein delivers localized planar velocity data on the streamwise centerplane, allowing direct identification of planar core flow structures.

However, existing studies have significant limitations in revealing the fine-scale flow features underlying this mechanism. The schlieren method employed by Skews provides only qualitative observations of the wavefront shape, lacking quantitative velocity data. Furthermore, subsequent PIV studies, such as that by Zhang et al. [29], achieved a temporal resolution of only 50 μs, which remains insufficient to capture microsecond-scale transient features. This study advances the PIV temporal resolution to 8 μs, enabling full-field velocity measurements of these transient interactions.

### 3.2. Flow Separation and Vortex Structure Evolution

**Figure 7:**
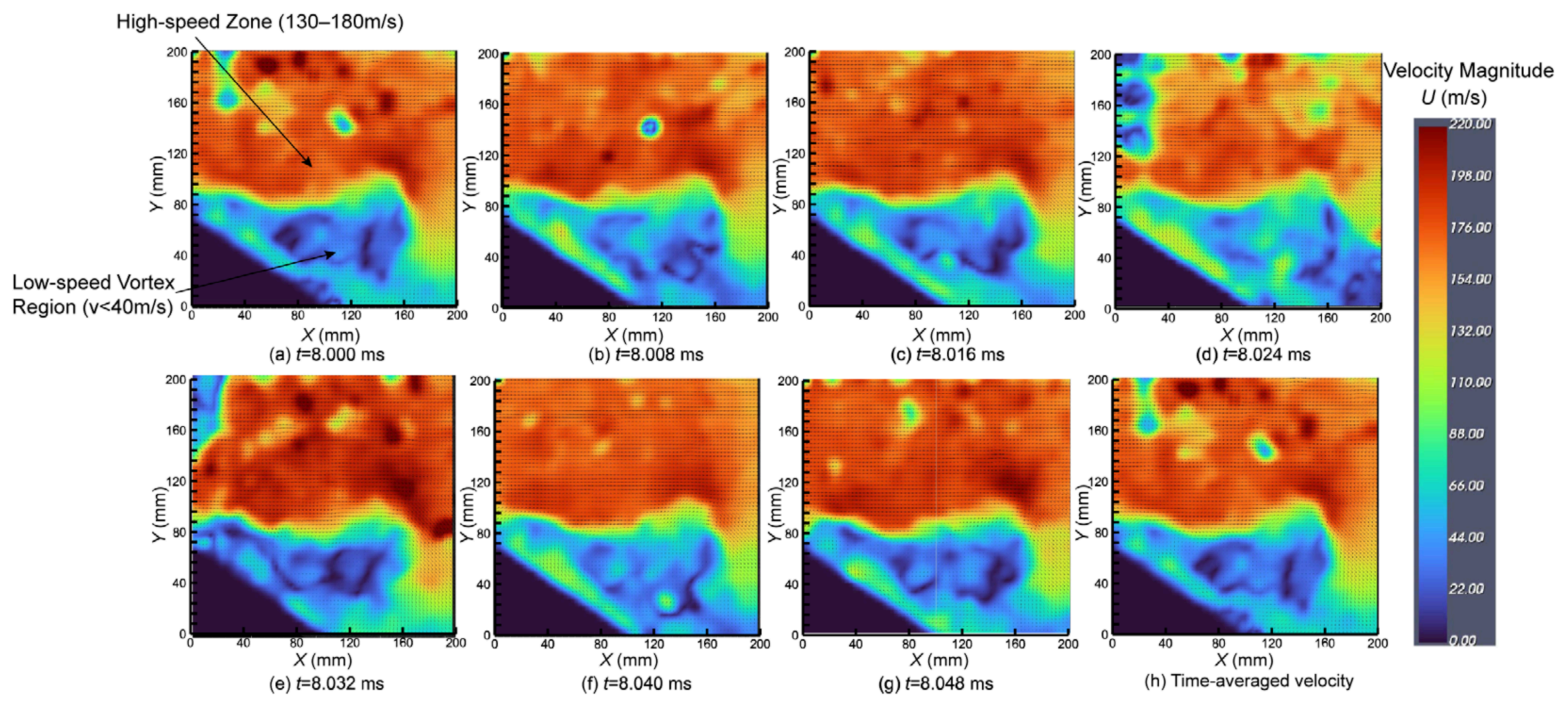
Instantaneous velocity field contour plot 8 ms after diaphragm rupture. The color contours represent the velocity magnitude $U=\sqrt{u^{2}+v^{2}}$ m/s.

The results above characterize the flow field as the shock wave traverses the observation window. As the shock propagates further downstream (*t* = 8 ms), the flow structure undergoes significant changes. The quantitative velocity fields (Figure 7: instantaneous contours and time-averaged field) reveal a large-scale primary separation vortex forming in the near-wall region downstream of the model. The vortex core remains spatially stable, and its shape is highly consistent across consecutive frames at 8 μs intervals. Furthermore, the velocity field exhibits significant non-uniformity. The outer edge of the vortex shear layer forms a local high-speed zone (130–180 m/s) driven by steep velocity gradients, whereas the vortex core constitutes a distinct low-velocity region (<40 m/s).

**Figure 8:**
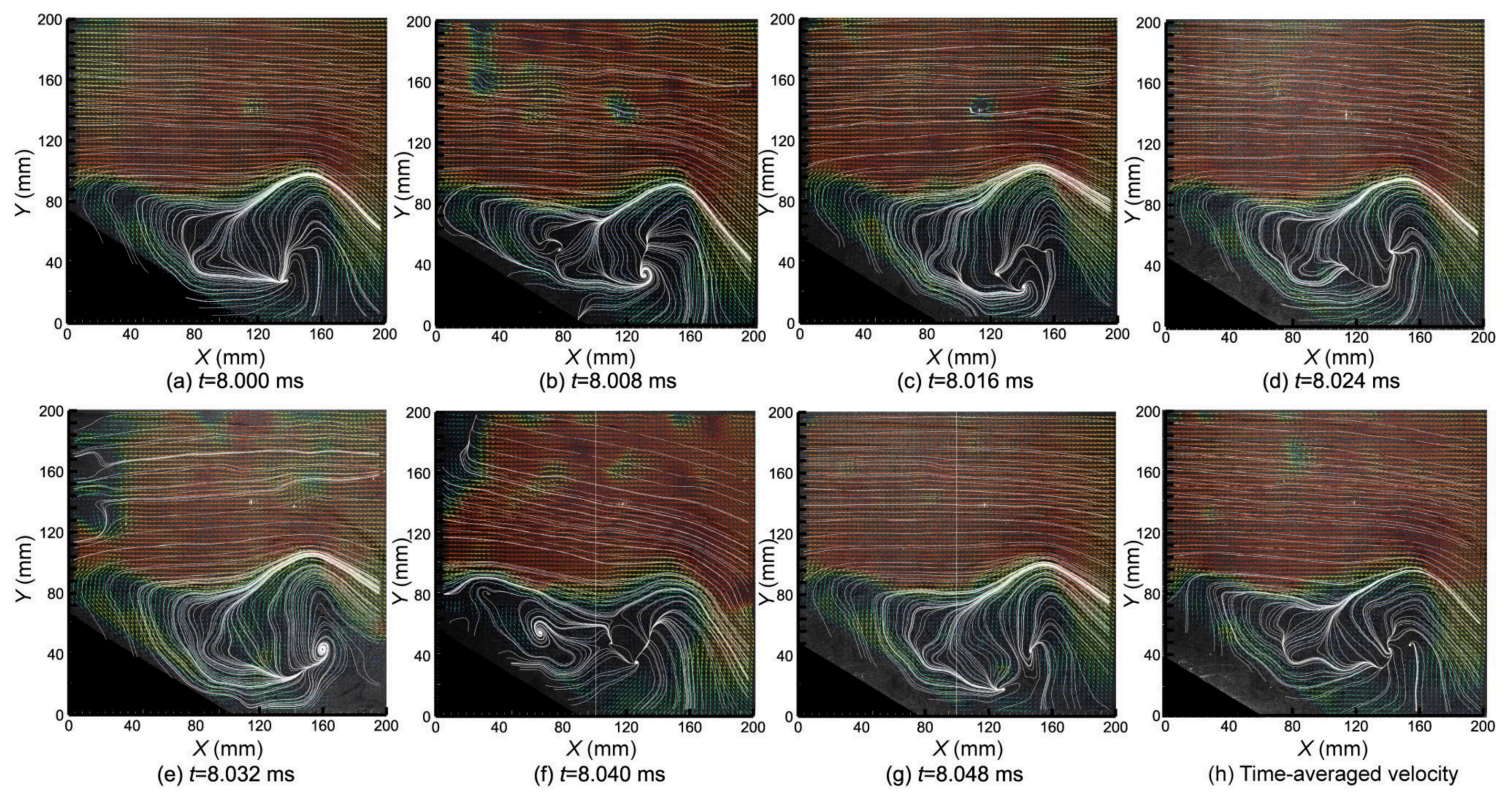
Instantaneous velocity field streamlines 8 ms after diaphragm rupture.

The flow field topology (Figure 8, instantaneous streamlines) clearly captures the roll-up and development of the vortex structure. The streamlines indicate that the primary vortex rotates clockwise, dictated by the direction of the shear layer velocity gradient. Throughout the continuous observations, this vortex structure maintains topological stability without obvious fragmentation or merging, indicating that the vortex evolution has entered a pseudo-steady state by *t* = 8 ms.

The primary vortex observed at *t* = 8 ms is a direct consequence of the flow state established during the pseudo-steady shock diffraction at *t* = 4 ms. Its core formation mechanism is the Kelvin–Helmholtz instability induced by strong velocity shear on the leeward side following shock diffraction [29]. Specifically, as the shock front passed at *t* = 4 ms, a significant velocity gradient formed on the leeward side. The flow velocity in the high-speed main stream was approximately 220 m/s, whereas the recirculation velocity on the leeward side was only about 30 m/s, creating a velocity difference of ~190 m/s.

(2) $$M_{c}=\frac{U_{1}-U_{2}}{a_{0}}=\frac{220-\left(\frac{220+30}{2}\right)}{340}\approx 0.28$$

The ability to accurately resolve such low-speed reverse flow within the recirculation zone—which typically suffers from low particle seeding density and severe background noise—directly benefits from the high SNR of the image frames. The strong cross-flow shear produces a convective Mach number $M_{c}\approx 0.28$, well within the low-subsonic domain of incompressible Kelvin–Helmholtz instability theory. This value falls just below the canonical compressibility threshold of $M_{c}=0.3$. As reported by Papamoschou and Roshko [30] and Slessor et al. [31], shear layer growth at this low $M_{c}$ differs from incompressible predictions by less than 5%, with negligible compressibility stabilization. This fully justifies the application of incompressible Kelvin–Helmholtz instability theory as a highly accurate physical approximation in this regime. Accordingly, the incompressible Kelvin–Helmholtz framework provides an excellent approximation to describe the present shear layer dynamics.

The sharp tangential velocity discontinuity between the high-speed outer stream and near-wall reverse recirculation provides the essential driving source for perturbation growth. According to linear stability theory, infinitesimal interfacial disturbances amplify exponentially at the most unstable wavelength, accompanied by continuous vorticity accumulation across the shear layer. Driven by such intrinsic unstable evolution, the shear layer progressively rolls inward and coalesces, ultimately forming the topologically stable primary vortex at *t* = 8 ms [32].

PIV velocity vector data show that the rotation direction of the primary vortex aligns with the shear layer velocity gradient, with the vortex core located in the region of strongest shear layer roll-up. This observation fully corroborates the classic physical evolution sequence consisting of shock diffraction, flow separation, shear layer formation, K–H instability, and eventual vortex roll-up in the wake flow behind an obstacle [10].

In their study on the interaction between shock waves and complex boundaries, Gichon et al. [10] proposed a dynamic model for vortex evolution by quantifying the relationship between shear layer thickness and vortex core strength. Their conclusions are consistent with the evolutionary characteristics of the primary vortex observed in this experiment, further confirming the reliability of our measurements. Notably, Gichon’s research primarily relied on numerical simulations and indirect measurements. In contrast, this study directly resolved the transient velocity field and flow topology using time-resolved PIV. This approach overcomes the limitations of classical experiments, which could only provide qualitative observations of vortex generation without resolving the underlying velocity structures.

### 3.3. Comparison of Numerical Simulations with Experimental Results

To establish a macroscopic reference for the flow field induced by the shock-trapezoidal obstacle interaction, numerical simulations based on the finite volume method were conducted concurrently. The computational domain replicates the experimental setup. It features a square shock tube with a 234 mm side length. A trapezoidal obstacle (75 mm high, 75 mm wide, and a 30° base angle) is placed at the bottom. The flow is governed by the three-dimensional unsteady compressible Reynolds-Averaged Navier–Stokes (URANS) equations. Following classical numerical studies of compressible shock-obstacle flows [33,34], the Realizable k-ε turbulence model with standard wall functions was employed. The working fluid is assumed to be an ideal gas. A density-based solver is utilized. Spatial fluxes are discretized using the AUSM+ scheme, and time advancement is handled by a second-order implicit scheme. A planar incident shock wave (*M*_a_ = 1.3) is generated by setting the pressure ratio between the high- and low-pressure regions. All walls are treated as adiabatic with no-slip conditions. A structured hexahedral mesh is adopted, with local refinements applied along the shock propagation path and near the obstacle. A grid convergence study was performed using three mesh resolutions: coarse (2.5 × 10^6^ cells), medium (5.0 × 10^6^ cells), and fine (1.0 × 10^7^ cells). The wall pressure profiles and shock front locations were compared across the three grids. The medium and fine grids showed differences of less than 2% in peak overpressure and less than 1% in shock arrival time, indicating that the medium grid achieves grid-independent results for the macroscopic flow features. All subsequent results are presented using the medium grid resolution.

The CFD grid cell size in the shear layer is approximately 0.15 mm, an order of magnitude smaller than the PIV vector spacing (1.35 mm). Despite this, the experimental velocity field resolves high-frequency fluctuations and small-scale vortex structures that are smoothed in the simulation. This confirms that the numerical dissipation is rooted in the over-viscous isotropic modeling of the URANS framework, not grid resolution limits.

**Figure 9:**
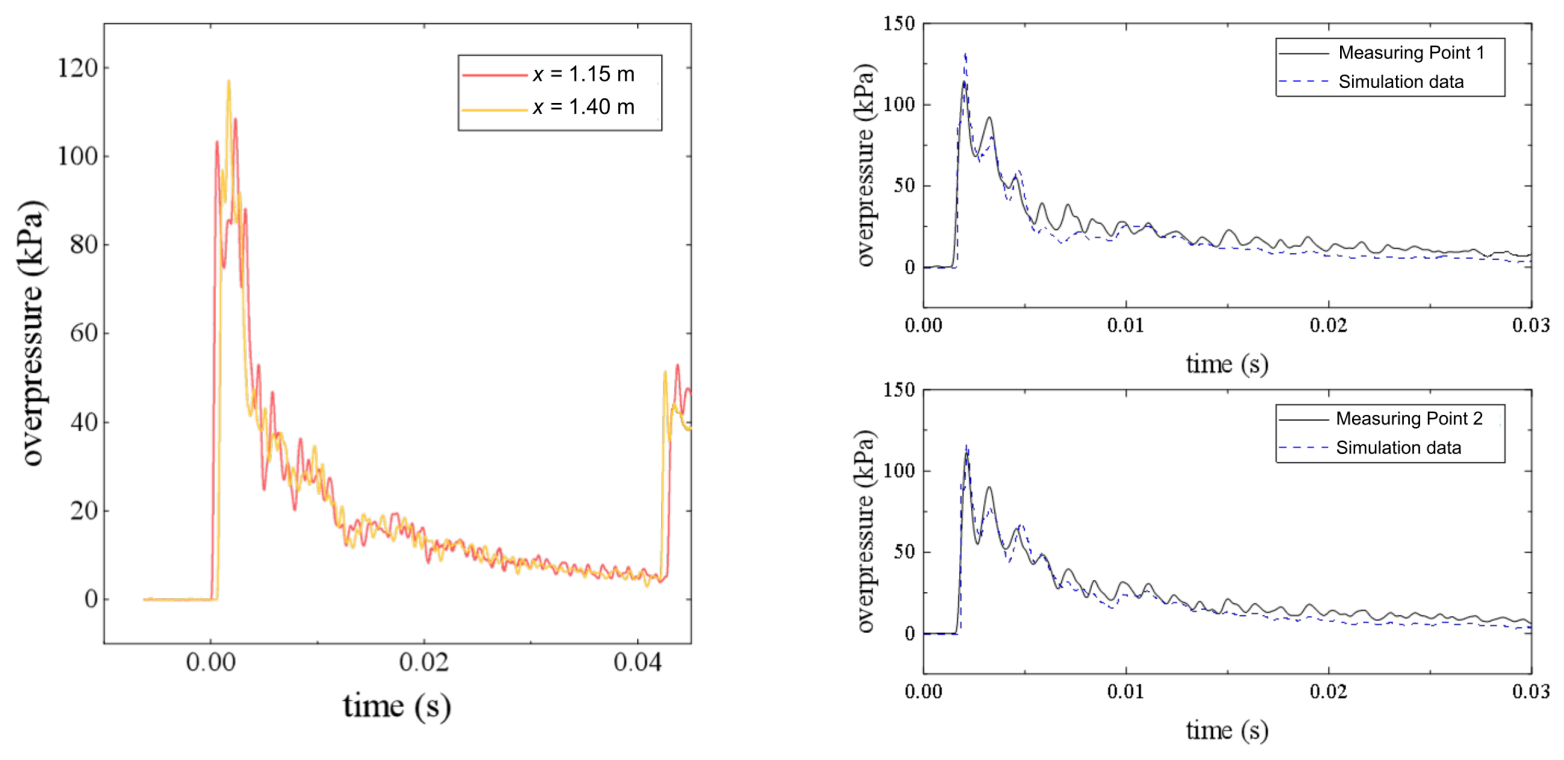
Comparison of measured and simulated pressure profiles.

As shown in Figure 9, previous tests examined the interaction between shock waves and complex terrain. In those tests, the calculated ground pressure profiles behind the slope matched the experimental results regarding shock propagation patterns and peak overpressure. This consistency indicates that the current URANS parameter settings are suitable for reproducing the macroscopic aerodynamic characteristics of such flows. In highly compressible flows, the pressure and velocity fields are strongly coupled. Therefore, the consistency of macroscopic pressure characteristics indirectly suggests that the computed velocity field can provide a reliable macroscopic reference for the flow field evolution.

**Figure 10:**
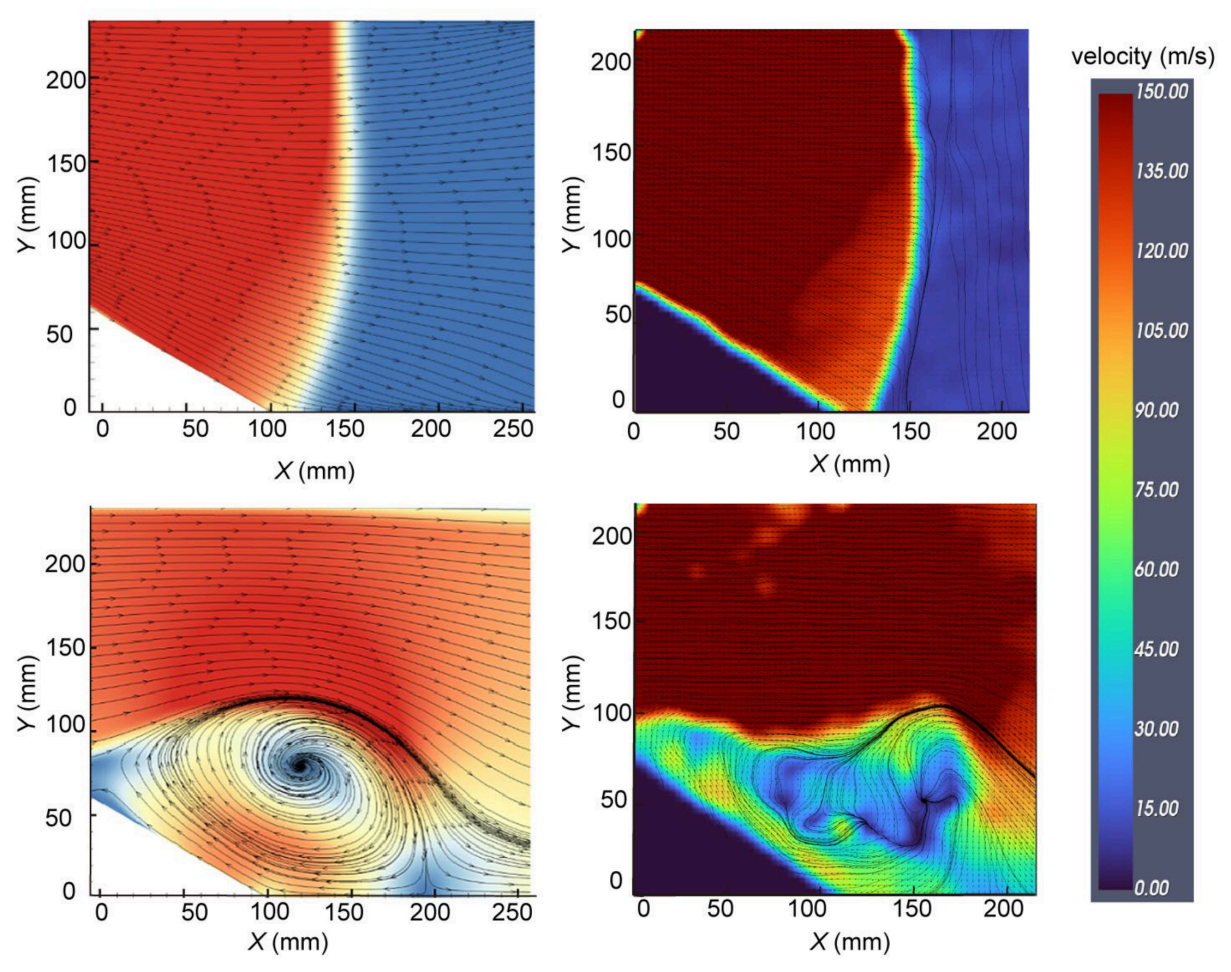
Comparison of measured and simulated velocity fields.

Visual comparison (Figure 10) exhibits good macroscopic qualitative agreement between the numerical simulations and PIV measurements. The velocity gradient distributions, velocity magnitudes, and vortex morphology, as well as the shock front location, flow separation extent, and spatial scale of the recirculation zone, all exhibit consistent trends.

Despite this macroscopic consistency, significant discrepancies emerge at the fine scale, reflecting the intrinsic gap between URANS-modeled turbulence and real transient flow physics. The nanosecond-duration laser pulses in the PIV system effectively freeze the particle motion, eliminating motion blur and allowing the precise capture of instantaneous flow structures. Consequently, these full-field transient measurements reveal fine-scale details that are smoothed out by the turbulence model, specifically manifested in the following three aspects:

1. Shock structural details. PIV not only resolves the sharp velocity discontinuities at the shock front—a region typically plagued by severe laser sheet reflections and particle imaging degradation, overcome here by the high-SNR frames—but also reveals complex local velocity gradient patterns in the near-wall region.
2. Vortex morphology. The simulation predicts a large-scale, stable vortex core with a regular shape in the recirculation zone. PIV, however, reveals dynamically evolving, spatially discrete clusters of small-scale vortices rather than a single regular core, reflecting the realistic complexity of vortex breakup and energy cascade processes.
3. Turbulent fluctuations. Subject to Reynolds averaging and numerical dissipation, the simulated velocity field is artificially smooth, filtering out small-scale fluctuations. The PIV-quantified field retains significant spatiotemporal fluctuations; specifically, streamlines at the shock front and shear layer exhibit highly complex, irregular branching structures characteristic of post-shock turbulence. Crucially, the high SNR of the acquired images ensures that these small-scale velocity fluctuations represent genuine physical turbulence rather than measurement noise, which typically obscures such fine-scale vortical structures.

These fine-scale discrepancies are fundamentally rooted in the nature of URANS. By solving statistically averaged equations with eddy viscosity models, URANS inherently outputs a time-averaged flow state; its isotropic assumptions and wall functions limit its capability to resolve anisotropic turbulence and near-wall unsteady effects, while numerical dissipation further suppresses unresolved fluctuations. In contrast, the microsecond-resolved PIV measurements capture the transient spatiotemporal evolution of the flow, successfully resolving key physical phenomena—such as instantaneous vortex dynamics and cross-scale intermittent momentum transport induced by shock–vortex interactions—that are completely smoothed out by the Reynolds averaging.

Therefore, the high-speed PIV results in this study not only provide a detailed physical picture of the shock–obstacle interaction, but the transient velocity fields and resolved turbulence characteristics also offer a reliable experimental benchmark for evaluating and improving the predictive capabilities of existing turbulence models.

## 4. Conclusions

This study developed and validated a parallel-channel particle image velocimetry (PIV) system with programmable timing control to resolve the inherent compromise between temporal resolution and illumination intensity in transient shock wave diagnostics. By distributing the imaging load across parallel channels, the system maintains a high single-pulse energy of approximately 400 mJ at 1 μs pulse separations, equivalent to a 1 MHz effective frame rate for an eight-frame burst. This diagnostic capability yielded motion-blur-free, high-SNR velocity fields of a planar shock wave interacting with a trapezoidal obstacle in a shock tube facility.

The time-resolved full-field velocity measurements captured the quasi-steady wavefront bending of the diffracted shock at *t* = 4 ms. This bending is driven by the spatial velocity gradient induced by Prandtl–Meyer expansion at the obstacle edge. At *t* = 8 ms, the system captured the subsequent flow separation and the roll-up of a topologically stable primary vortex. The calculated low convective Mach number (*M_c_* ≈ 0.28) confirmed the rapid development of classical Kelvin–Helmholtz instabilities in the wake region. These findings clarify the physical coupling mechanism: shock diffraction generates spatial velocity gradients, which subsequently trigger shear layer instability and vortex roll-up.

Comparative analysis with URANS simulations (validated by wall pressure history) revealed limitations in the numerical model. While it predicted the macroscopic shock structure and vortex shedding phase, it could not resolve fine-scale post-shock disturbances, dynamic vortex breakup, or anisotropic turbulent fluctuations. The PIV measurements directly visualized these small-scale features, highlighting the artificial smoothing effects of Reynolds averaging and numerical dissipation. These results establish the developed high-speed PIV system as a precise experimental benchmark for validating advanced numerical frameworks (such as LES and DNS). Furthermore, the system demonstrates potential as a diagnostic tool for investigating passive blast protection and terrain-induced energy dissipation in complex environments.

The parallel-channel PIV system achieves high accuracy in resolving transient shock-tube flow fields. Nevertheless, several practical limitations must be acknowledged: cumbersome alignment and calibration workflows for eight coaxial laser beams and cameras, linearly rising hardware costs associated with additional laser cavities and CCD detectors, and geometric and polarization-combination constraints that impede scaling beyond 8–12 measurement channels. Future efforts will streamline the optical assembly and hardware setup to boost system portability and cut operational complexity. Such improvements will extend the scope of this diagnostic technique to complex transient flows, blast-driven flow events, and unsteady fluid–structure interactions, delivering robust experimental benchmarks for validating computational simulations.

## Figures and Tables

**Table 1:** Main parameters of the parallel-channel PIV system.

Category	Parameter	Value/Description
Illumination and Imaging System	Laser type and wavelength	8-cavity Nd:YAG laser, λ = 532 nm
	Single-pulse energy	400 mJ/pulse
	Camera and resolution	CCD camera, 2367 × 2254 pixels (after dewarping)
Particle Seeder	Tracer particle type and particle sizes	Oil-based fog tracer particles, approximately 5 μm in diameter
Optical Configuration and Field of View	Optical thickness	approximately 1 mm
	Measurement plane	X-Y plane
	Field of View (FOV)	200 mm × 200 mm
	Spatial resolution	0.0845 mm/pixel

## Data Availability

The data presented in this study are available on request from the corresponding author. The raw particle image data and processed velocity fields are not publicly available due to institutional data management policies but can be shared for academic collaboration upon reasonable request.
